# Climate Change, Sea-Level Rise and Implications for Coastal and Estuarine Shoreline Management with Particular Reference to the Ecology of Intertidal Benthic Macrofauna in NW Europe

**DOI:** 10.3390/biology1030597

**Published:** 2012-11-05

**Authors:** Toyonobu Fujii

**Affiliations:** Environment Department, University of York, Heslington, York YO10 5DD, UK; Email: t.fujii@abdn.ac.uk; Tel.: +44-1224-274430; Fax: +44-1224-274402

**Keywords:** sea-level rise, coastal squeeze, benthic macrofauna, shorebirds, fish, shoreline management, estuarine ecosystems, managed realignment

## Abstract

In many European estuaries, extensive areas of intertidal habitats consist of bare mudflats and sandflats that harbour a very high abundance and biomass of macrobenthic invertebrates. The high stocks of macrobenthos in turn provide important food sources for the higher trophic levels such as fish and shorebirds. Climate change and associated sea-level rise will have potential to cause changes in coastal and estuarine physical properties in a number of ways and thereby influence the ecology of estuarine dependent organisms. Although the mechanisms involved in biological responses resulting from such environmental changes are complex, the ecological effects are likely to be significant for the estuarine benthic macrofauna and hence the consumers they support. This paper reviews the utilisation patterns of estuarine intertidal habitats by shorebirds, fish and crustaceans, as well as factors affecting the distribution, abundance and biomass of estuarine macrobenthos that is known to be important food source for these estuarine predators. This study also provides simple conceptual models of the likely impacts of sea-level rise on the physical and biological elements of estuarine intertidal habitats, and implications of these results are discussed in the context of sustainable long term flood and coastal management in estuarine environments.

## 1. Introduction

Estuaries are ecologically important coastal environments situated between freshwater rivers and the sea, characterized by highly varying physicochemical, morphological and hydrological conditions [1,2], which exhibits some of the most biologically productive habitats on earth [3,4]. These habitats have long been known as important nurseries for species of fish and crustaceans [5,6,7], and estuarine intertidal sand- and mud-flats are of great conservation value because they often serve as vital feeding grounds for many wintering shorebirds [8,9,10,11]. Many of the world’s estuaries are, however, already significantly affected by anthropogenic activities, by virtue of their long history of usage for human settlement, for agricultural and industrial development, for navigation, trade and transportation, and for biological exploitation. Estuaries have also been used as repositories for the effluent of industrial processes and domestic waste [4], and also prime sites for land reclamation for industrial or agricultural lands [4,5]. They are also natural sinks for contaminants, such as agricultural biocides and nutrients, originating from the catchment hinterland [3,12]. Estuaries and their fringing wetlands or low-lying hinterlands are important habitats for wildlife, but at the same time under ever increasing pressure from anthropogenic activities, and they are likely to continue to experience a diverse range of environmental stressors such as habitat loss, habitat alteration, eutrophication, overfishing and freshwater diversion. Furthermore, warming of the global climate system is now evident with the linear warming trend over the 50 years between 1956 and 2005 nearing twice that for the 100 year between 1906 and 2005 [13] and this is likely to cause the present rate of sea-level rise to increase [14], posing additional concerns for coastal and estuarine environments. Amongst the most serious issues relating to climate change are increased flood risks resulting from rising sea levels, threats to the maintenance of coastal hard defences induced by increased wave and tidal energy and the process of coastal squeeze by which the area of intertidal habitats is reduced as the beach is prevented from migrating inshore due to the physical presence of hard coastal defences. Sea-level rise will therefore undermine the welfare of both wildlife and human society around estuarine environments, so that management decisions will have major implications for the health of both estuarine ecology and society.

This paper considers the implications of climate change and associated sea level-rise for estuarine and coastal management on time scales of decades up to a century (medium- to long-term) with particular reference to the ecology of macrofauna on an estuarine intertidal habitat. This study first reviews the utilisation patterns of estuaries by shorebirds, fish and crustaceans, as well as factors affecting the distribution, abundance and biomass of estuarine intertidal macrobenthos that is an important food source for those predators. This is followed by a brief account of the most likely sea-level rise scenarios and an account of the likely impacts on the physical elements of coastal and estuarine ecosystems. Simple conceptual models are then presented to consider the likely impacts of climate change and sea-level rise on the physical and biological properties of estuarine intertidal habitats with reference to a case study of macrobenthic biomass in relation to changes in sediment particle size, beach morphology and salinity regime in the Humber estuary, UK. How changes in other environmental parameters such as ambient temperatures or nutrient loading may interact with the effects of sea-level rise to shape the structure and function of invertebrate assemblages is also considered. Finally, the adaptive strategies likely to be adopted to cope with sea-level rise are discussed and implication is given as to how to identify appropriate ecological objectives for sustainable shoreline schemes in estuarine environment in the future.

## 2. Estuarine Benthic Macrofauna and Their Predators

In coastal and estuarine ecosystems, birds are often regarded as top predators and fish occupy intermediate trophic levels, both of which are supported by a large amount of intertidal benthic macrofaunal prey. The high benthic biomass is a reflection of the presence of extensive intertidal flats and fine sedimentary deposits created through processes such as tidal asymmetry and flocculation [15]. Typically, there are coarser deposits on relatively exposed areas around the outer reaches and finer particles in sheltered areas as well as in the upper reaches of estuaries, depending on the local hydrographic regime and chemical processes. Fine muds and silts provide a large surface area for the accumulation of organic matter and microbial processes, which in turn allows intertidal mudflats to support a high invertebrate biomass, especially with deposit- and filter-feeding taxa [15,16,17]. This benthic invertebrate biomass provides food for higher trophic levels, such as epibenthic crustaceans, fishes and shorebirds, which use the intertidal flats as a nursery area for juvenile stages and/or as adult feeding grounds [4].

Much of the interest from conservation and management agencies in evaluating the effects of sea-level rise on estuaries lies in the potential impact on these higher trophic levels, especially migrating shorebirds [18,19]. In NW Europe, particularly dependent avian species are brent geese, shelduck, pintail, oystercatcher, ringed plover, grey plover, bar-tailed and black-tailed godwits, curlew, redshank, knot, dunlin and sanderling, whilst grey geese and whooper swan may utilise this habitat for roosting [18,20]. The prey base for these avian species can be narrow with well over 90% of the total benthic macrofauna comprising a relatively small range of species [8,21]. Except for some herbivorous and omnivorous species such as brent goose, mallard or golden plover, many of shorebirds feed mainly on intertidal organisms when the flats are exposed, and they can be highly selective both as to where they feed and the size of prey, in order to maximise their efficiency [22]. Differences in food availability account for a large proportion of the variation in densities of bird feeding in intertidal habitats [10,23], and shorebirds tend to concentrate where prey density and availability are relatively high and the energy expenditure required to feed is relatively low [24]. Intertidal flats within an estuary exhibit significant spatial variations in macrofaunal species composition, density and biomass, and this is highly correlated with substratum type or sediment particle size [25]. Thus, Yates and colleagues [10] were able to show that broad sediment characteristics can be used to predict the densities of shorebirds, allowing bird distributions to be predicted from sediment maps derived from remote sensing data. If the size of feeding ground and the distribution of sediment types change with increasing sea levels, there is likely to be a response in bird distribution and abundance. This has indeed been the case where change in substratum type or loss of intertidal habitat has occurred. For instance, the spread of the cordgrass, *Spartina anglica*, over the mud flats in southern England resulted in reductions in the numbers of Dunlin using affected areas [26]. Similarly, due to the impact of habitat loss as a result of land-claim on the intertidal area of the Forth estuary, Scotland, significant declines were noted for overwintering populations of dunlin and bar-tailed godwit [5]. Sea levels are rising worldwide and any reduction in local feeding habitats due to coastal squeeze therefore could have a synchronising impact on overall shorebird populations globally [27].

Many fish and crustaceans also utilise the intertidal flats with the flood tide to feed [4,7]. A diverse range of fish is found in many estuaries and the majority are bottom feeders [7]. Fish have the option of feeding intertidally or subtidally, but there has been an increasing evidence for the importance of intertidal feeding for many of fish species either throughout life or in part of their life cycle in estuarine environments [6,28,29,30]. In summer in Europe, for example, large numbers of flatfish (*Playtchthys flesus*, *Pleuronectes platessa*), gobies (*Pomatoschistus* spp.), crabs (*Carcinus maenas*) and shrimp (*Crangon* spp.), move onto the flats to feed on mobile epifauna and sedentary infauna [20]. They also crop parts of prey, such as the tail ends of *Arenicola* and *Heteromastus*, the feeding tentacular crowns of fanworms and siphons of bivalve molluscs [22,31]. Even piscivorous species such as cod *Gadus morhua* or whiting *Merlangius merlangus* are heavily dependent upon estuarine macrobenthos through direct feeding on benthic infauna, or through feeding on other fish species, crab *C. maenas*, shrimp *C. crangon* which have themselves fed on the benthic invertebrates [32]. Migratory species, such as salmon and eels, can also be found in these areas on passage to other wetlands, although they appear to have no requirement for mud and sand intertidal flats [20]. With respect to prey preference, many demersal fish are opportunistic predators within estuarine environments and the choice tends to reflect the distribution patterns of infaunal species in the area [30]. In tropical and subtropical areas, the juveniles of many species of crustaceans of commercial importance, particularly penaeid prawns, utilise estuaries, lagoons and mangroves for feeding grounds before migrating offshore to spawn [15]. In view of the large number of juvenile fish and crustaceans observed, estuaries are potentially important in the maintenance of commercial offshore fisheries. For instance, it has been argued that five out of the six most important commercial fishery species in the USA may be dependent upon estuaries [33] and extensive estuaries on the east coast of the USA may be responsible for at least half the commercial landings each year [34]. Some lessons may be learned from coastal and estuarine systems in Nigeria where intensive human activities such as land-claims and heavy pollution have severely affected the breeding and nursery grounds of commercial fish species, and landings from capture fisheries have declined from 500,000 t in the late 1980s to 300,000 t in the early 1990s [35].

Degree of dependence on estuarine benthic macrofauna can also be measured by predation pressure from those fish, crustaceans and shorebirds. For instance, redshank are estimated to remove 16%–38% of intertidal amphipod crustacean, *Corophium voltator*, on the Ythan estuary, Scotland [36], bar-tailed godwits 25% of lugworm, *Arenicola marina* [37], and oystercatchers 14% of the mussels, cockles and other molluscs [38] on which they prey, although the impact of birds on intertidal invertebrates varies greatly from site to site [9]. Food consumption of fish that move into estuaries at various seasons also has an impact roughly equal to or even higher than that of the birds. For instance, plaice and flounder consume up to 15 g ash-free dry weight of benthos m^−2^ yr^−1^, which accounts for 30% of the estimates of total benthic production, in the Oosterschelde in the Netherlands, whereas in Ythan estuary in Scotland, the fish are estimated to consume three times the amount of food consumed by the birds [4]. Given the high degree of dependence on the standing stock of the estuarine macrobenthic invertebrates, any change in their biomass and production is likely to impact on predator populations, and this will be especially so for the more productive intertidal, as compared to subtidal areas. An understanding of the likely responses of invertebrate assemblages to changes in key environmental elements associated with sea-level rise is therefore of paramount importance in estuarine ecosystems.

## 3. Effects of Sediments and Intertidal Morphology on Estuarine Intertidal Benthic Macrofauna

### 3.1. Exposed Estuarine Shores and Sandy Intertidal Flats

Exposed shores and sandy intertidal flats are often located at the outer region of an estuary. The overall morphology and dynamics of the beach, captured by the concept of “dissipative” (with flatter slope, low energy conditions and finer sediment) and “reflective” (with steeper slope, higher energy conditions and coarser sediment) spectrum, is one of the predominant factors in controlling the distribution and abundance of intertidal faunal assemblages on these exposed sandy intertidal flats [39]. For instance, on the north and west coasts of Scotland, McIntyre [40] found that on exposed sandy flats, isopods, such as *Eurydice pulchra*, dominated, but with moderating exposure, the proportion of polychaetes increased. The highest biomass was found on sheltered beaches dominated by molluscs, mainly the bivalve *Tellina tennuis*. Similarly, McLachlan [41] reviewed Australian, South African and north-east Pacific USA beaches and found that species diversity showed a linear increase from reflective to dissipative beach state and from steep to flat slopes with abundance increasing logarithmically. However, biomass was best correlated with wave energy rather than beach morphodynamic state, whereas individual biomass increased with exposure, suggesting the total faunal biomass on exposed and semi-exposed beaches may not differ as much as expected [42]. In NW Europe, mussel beds (*Mytilus edulis*) can form extensive biogenic reefs with substantial biomass on at lower shore levels, particularly at the mouths of meso- and macro-tidal estuaries where there is favourable tidal flow [43]. Except for such patchy areas of high biomass, the assemblages found in exposed estuarine sandy areas are generally characterised by a high diversity in types of organisms but neither a high biomass nor a high productivity. With decreasing exposure and increasing sediment stability, species richness, abundance and total biomass increase to reach a maximum on muddy sheltered shores.

### 3.2. Sheltered Estuarine Shores and Mud Flats

Sheltered shores are found in areas of low energy and they have poorly sorted sediments with high levels of organic matter and a high silt content [44]. Fine particles tend to accumulate at higher shore levels and particle sizes become sandier towards lower shore levels to form coarse mobile sands in the subtidal river bed due to scour. The levels of biomass and production of estuarine benthic macrofauna within the mudflats are typically much higher than in subtidal areas [4,5,45] and rates of annual production may differ by a factor of two [16]. This high biomass and production is characteristically attributable to the large number of deposit- and filter-feeding invertebrates. Thus polychaete worms such as *Nerine* and *Ophelia* become more frequent in area of moderate disturbance, and with more sheltered conditions, burrowing bivalve molluscs, such as tellinids and large clams, and epibenthic mussels become abundant [15]. In addition, several tidal migrants occur including mysids, amphipods and decapods or drifting species associated with algal growths (e.g., *Neomysis integer*, *Melita obtusata*, *Dexamine spinosa*, *Stenothoe marina*, *Idotea* spp.).

The nature of the substratum and tidal level can markedly affect the distribution patterns of benthic macrofauna within an intertidal area [4]. In Britain, for instance, Eltringham [46] observed a three-zone pattern on muddy shores: an upper zone characterized by either a complete absence of fauna or by the polychaete *Nereis diversicolor*; a mid-tide zone of the bivalve molluscs *Scrobicularia plana*, *Cerastoderma edule* and *Macoma balthica*, and where some sand is present, the lugworm *Arenicola marina*; a low-shore zone with the bivalves *Mya arenaria* and possibly *Tellina fabula*, depending on the sediment particle size. The relationships between tidal level and abundance or biomass of macrofauna tend to show similar patterns between tidal flats in different locations. For instance, in different parts of the Dutch Wadden Sea, Beukema [47] showed that numerical abundance and biomass of macrofauna increased from values close to 0 at high water level to maximum values around mean-tide level or halfway between this level and low-tide level, then declined to low values towards the low water level, whereas mean biomass per individual increased from high- to low-water level. A similar trend was shown on intertidal flats in the Humber estuary, UK, where relations between relative macrobenthic biomass and tidal level exhibited a concave quadratic pattern with the biomass attaining the highest value where the tidal depth was located approximately 40% of the tidal range lower than mean high water level [25]. It is clear that tidal height and sediment type can play an important role in shaping the distribution and abundance of the benthic macrofaunal assemblages in estuarine intertidal flats.

## 4. Effects of Salinity on Estuarine Intertidal Benthic Macrofauna

In contrast to studies of macrofaunal biomass and production, many publications on the distribution range of species and the diversity of benthic assemblages in estuaries have focused on salinity gradient [16,48,49]. Diversity generally declines on shores affected by low salinity [45,50,51,52], and in the case of Severn estuary UK, the body size of the lugworm *Arenicola marina* also diminishes with distance from the sea [53]. It has been suggested that the fauna can be divided into a series of communities with distance from the mouth up-estuary [54], and Little [55] points out that many of the communities in the outer regions are related to sediment types, hydrology and depth, but in the inner estuarine areas, the very low diversities correlate with low salinity. Furthermore, in those studies where biomass over the entire estuarine longitudinal (salinity) gradient has been described, there is a trend from lower biomass in the upper estuarine regions to higher biomass in the more downstream (seaward) parts [25,45,50,56,57]. With respect to productivity, Edgar and Barrett [58] showed that faunal biomass and estimated productivity were highly correlated with salinity at the low-tide and shallow subtidal level for Tasmanian estuaries. Salinity seems therefore to be a key determinant of intertidal macrofaunal abundance and distribution patterns along estuarine longitudinal gradient. 

## 5. Effects of Climatic Conditions and Temperature on Estuarine Intertidal Benthic Macrofauna

Inter-annual variability in climatic conditions will have strong influence on temporal variability of estuarine macrobenthic assemblages through changes in individual growth rates, fecundity and recruitment success. In the long-term study in the Wadden Sea in the Netherlands, Beukema and colleagues [59] noted that year-to-year fluctuations were marked for almost all benthic species, which were known to be important food sources for higher trophic levels, and in the Balgzand area, 12 out of 29 species showed higher rates of mortality during cold than during mild winters, and several characteristic species such as *Cerastoderma edule*, *Nephtys hombergii*, and *Lanice conchilega*, showed minimal biomass values due to low over-winter survival during severe winters. In addition, bivalve species such as *Macoma balthica* show a significant negative correlation between winter temperatures and subsequent recruitment, with higher *M. balthica* densities in the year following a cold winter, probably due to an increase in fecundity [43,59,60]. Predatory juvenile fish and crustaceans can also be affected by the severe winters, and it is notable that recruitment of the cockle *Cerastoderma edule* is unusually high in the summers following widespread mortality of the consumers of their spat [61]. Changes in ambient temperature will also alter individual growth rates and other physiological functions of benthic organisms in estuarine environments. For example, Moens and Vincx [62] investigated respiration and food assimilation of two estuarine nematodes at a range of temperatures, salinities and food densities, and found that temperature had a large effect on metabolic rate and production, showing increase with higher temperature up to 25 °C. Secondary production in benthic invertebrates can be estimated directly, but such estimates are usually taxa-dependent, time-consuming and labour-intensive [63]. Indirect estimates based on empirical relations are available [64] and the latter reveals a strong positive effect of warmer temperature on secondary production. The effects of climate change on intertidal macrobenthos will be complex at species level, but the possibility of milder winters and warmer summers in the future implies that the overall biomass of estuarine benthic assemblages may be expected to increase. As yet it has not been possible to determine the degree to which any loss of invertebrate biomass resulting from sea-level rise and associated coastal squeeze may be compensated by these factors.

## 6. Effects of Food Supply on Estuarine Intertidal Benthic Macrofauna

There is an increasing body of evidence which to confirm food supply regulates benthic biomass and secondary production by affecting individual growth and fecundity of adult invertebrates at the estuarine system scales [16,17,65]. For instance, Heip and colleagues [16] examined the relationships between pelagic primary production, advection of organic matter, sedimentation of organic carbon, and benthic biomass and productivity, and found a significant dependence of total system biomass of commercial benthic suspension feeders on the residence time of the water in the system. The model assumed that residence time was an inverse measure of food exchange with the coastal sea, and that system productivity was the basic limiting factor for suspension feeder biomass which is often the dominant component of estuarine benthic assemblages. In the case of Balgzand area of the Dutch Wadden Sea, Beukema and Cadée [66] showed that a substantial increase of pelagic primary production between 1970s and the 1980s was followed by a nearly proportional increase of system-averaged benthic biomass. Similarly, in a comparison of 15 different estuarine systems worldwide, Herman and colleagues [17] found that system-averaged macrofaunal biomass was significantly correlated with the system primary production. However, primary production itself is controlled by such factors as light, temperature [67], nutrients [16], and anthropogenic activities (e.g., discharge, dredging) [68]. It is therefore desirable to understand how in the long term climate change and associated sea-level rise will affect any of these factors, which in turn regulate primary productivity and hence long-term change in total macrofaunal biomass within an estuary at the whole system scales.

## 7. Climate Change and Sea-Level Rise Scenarios

The Earth’s climate has warmed by 0.6 ± 0.2 °C during the last century (1901–2000) [13] and research in recent years has demonstrated that human activities, mainly through the emission of greenhouse gases or aerosol particles, are likely to be responsible for the observed increases in contemporary climate changes [13]. 2000–2009 was the warmest decade on record [14], and evidence for warming is now unequivocally seen for other physical and ecological indicators, such as retreating glaciers, thinning of Arctic sea-ice [69,70], changes in the phenological behaviour and/or shifts in the habitat ranges of various terrestrial and marine species [71,72].

Sea-level changes reflect climate change mainly through thermal expansion of the upper layer of the oceans and release of water from glacier or ice sheet melt [69,70]. Sea-level change is a natural process at the geological time scale, showing a marked fluctuation pattern over a range of 120 m during the last 140,000 years [73]. While a continuous rise of sea level has been observed since the last de-glaciation approximately 19,000 years before present [73], contemporary trend shows global average sea level rose at an average rate of 1.8 mm per year between 1961 and 2003 whereas this rate was faster at an average rate of about 3.1 mm per year between 1993 to 2003 [13].

At the global scale, models predict that the annual global mean surface air temperature will rise 1.1–6.4 °C by 2100 based on increases of greenhouse gases [13], and the recent climate models estimated sea level rise projections of 57–110 cm by 2100 [74]. The wide range of estimates reflects the full set of the scenarios analysed based on expectations of changes in emission of greenhouse gases and pollution of the atmosphere as well as degree of mitigation [74]. Therefore the amount by which sea levels will rise is dependent upon the scenarios, and the relationships diverge with time in a non-linear manner with the most realistic estimate of around 74 cm by 2100.

However, there are likely to be marked regional and local-scale variations relating to sea-level rise [13,75,76], and estuarine and coastal environments are thus subjected to be exposed to different levels and magnitudes of risks induced by climate change. For example, the rate of sea-level rise relative to land will vary depending on geological location, due to a constant isostatic readjustment of the height of land masses caused by changing relationships between the volumes of water in the oceans and the amount of land-based ice [73]. Thus much of southern England is submerging at the rate of between 1.0 mm and 1.5 mm per year relative to the sea, while much of Scotland is emerging at between 0.5 mm and 1.0 mm, in the UK [77]. Therefore, it is necessary to take account of the adjusted net rates of sea-level rise, and in the case of the Humber estuary UK, for example, an average rate of sea-level rise is assumed to be 6.0 mm per year over this century for planning purposes, compared with 2.0 mm per year over the last century [78].

In addition to global warming and increased sea level, there are predicted changes in the frequency of severe droughts, excessive precipitation and extreme events together with changes in seasonal and diurnal temperatures at local and regional scales [13,77]. These aspects of climate change also have important implications for estuarine and coastal management because coastal areas are likely to experience a higher frequency of storm surges due to extreme climatic events, superimposed on the more gradual sea-level rise. The return period of surges scales logarithmically with the rising water levels [77] and, as a result, a relatively modest rise in sea level increases the frequency of surges and thus the risk of coastal flooding markedly. In addition, the energy of waves reaching intertidal flats is a function of both local water depth [79] and the height of the waves offshore [1]. For coastlines where an increase in average wind-speed and wind extremes is predicted, waves will be higher and the wave climate over intertidal habitats will be more energetic. If sea-level rise results in deeper water locally, then this will exacerbate the problem, because rates of beach erosion will increase and less wave energy will be dissipated prior to the wave breaking on the shore. Therefore, in addition to a straightforward loss of intertidal habitats, sea-level rise is likely to undermine the infrastructure of coastal defences requiring the increase in maintenance costs of the structures.

## 8. Implications for Estuarine Physical Elements

There are several estuarine physical processes in response to sea-level rise that are of great relevance for the management of estuarine ecosystems. Rising sea level will result in changes in coastal and estuarine geomorphology as a consequence of increased water depth and enhanced wave and tidal energy [79]. Such physical changes will be manifested through the landward progression or redistribution of landforms such as subtidal bedforms, intertidal flats, salt marshes, shingle banks, sand dunes, cliffs and low-lying hinterland [80]. Thus, the extensive linear sand-banking, which is formed by dominant marine processes and often seen in the lower estuary, is expected to migrate upstream in response to rising sea level. Similarly, intertidal mudflats that are found in less exposed environments will also migrate landwards to a lower energy level as coastal wave energy increases [81]. If the estuary is allowed to migrate inland, then it could re-establish its original structure further upstream. However, for many estuaries this will not be permitted due to existing flood defences protecting agricultural and residential areas and due to canalisation especially further upstream. This inability for the estuary to move inland and upstream will lead to substantial losses of intertidal habitats due to coastal squeeze, a process by which salt marshes and mudflats are eroded away as they become trapped between rising sea-levels and fixed sea defences. This reduction in spatial extent of intertidal habitats would inevitably decrease the amount of benthic intertidal invertebrates on which larger consumers, such as wintering shorebirds, fish and epibenthic crustaceans, are dependent. If land is made available above the current high water level for the replacement of important estuarine intertidal habitats by setting back current flood defences (“managed realignment”), this will reduce the environmental impacts in estuarine ecosystems. However, managed realignment may not be a viable option for most estuaries because of the economic value of the bordering land.

In addition to the loss of habitat due to coastal squeeze, change in sedimentation and geomorphological processes over intertidal flats in response to sea-level rise will have important implications for the estuarine ecosystem. Estuarine intertidal mudflats with fine muddy sediment and a rich organic matter content typically support high numbers of benthic invertebrates, which in turn plays a central role in maintaining estuarine food webs [4,55]. Through natural sedimentary processes in response to sea-level rise, mudflats will migrate landwards and could be replaced by sand beaches, which have similarly migrated from more exposed coastal environments [81], shifting the entire sedimentary distribution both orthogonal and parallel with estuarine longitudinal shorelines. However, existing flood defences are likely to prevent the re-establishment of mudflats at higher shore, whilst increased water depths and a deteriorating wave climate may lead to changes in the beach’s morphodynamic state moving from a dissipative beach to a reflective one. Such shifts may be more pronounced in areas around the outer reach of an estuary where wave action and extreme climatic events are dominating forces in the process of coastal land formation. For instance, Taylor and colleagues [82] have investigated changes in 1084 coastal profiles throughout England and Wales, and found that 61% of the coastlines studied had experienced steepening since in the middle of 19th century, generally due to foreshore erosion and the use of sea walls and embankments.

Finally, there will be effects of sea-level rise on the estuarine salinity gradient from freshwater to marine conditions. The structure of this gradient varies according to estuarine morphology, freshwater run-off and turbulence or mixing [1,15]. Many of world’s estuaries will experience a widening and deepening in estuarine water volume resulting from rising sea levels and a concurrent increase in tidal prism and tidal range [3], leading to a greater salt intrusion further upstream. This upstream shift in salinity gradient may affect the vegetation communities fringing the estuary through penetration of salt water into the fresh ground water table, leading to salinisation of habitats currently characterised by freshwater [73]. In addition, shifts in salinity distributions will result in changes in the species composition of benthic communities, as well as shifting the region of turbidity maximum, where vigorous mixing of fresh and marine water and intensive particle deposition occur, further upstream. This would increase local silt accretion rates further upstream, but reducing silting processes and increasing the accumulation of coarser sediment in the outer reaches of the estuary.

These general processes will be modified by local geology, the land use of drainage basin, the size, shape and human usage of the estuary [73], and it is therefore important to carefully examine the physical settings of individual estuary to predict the impacts of sea-level rise on these ecosystems.

## 9. Implications of Sea-Level Rise for Estuarine Macrobenthos and Shoreline Management

**Figure 1:**
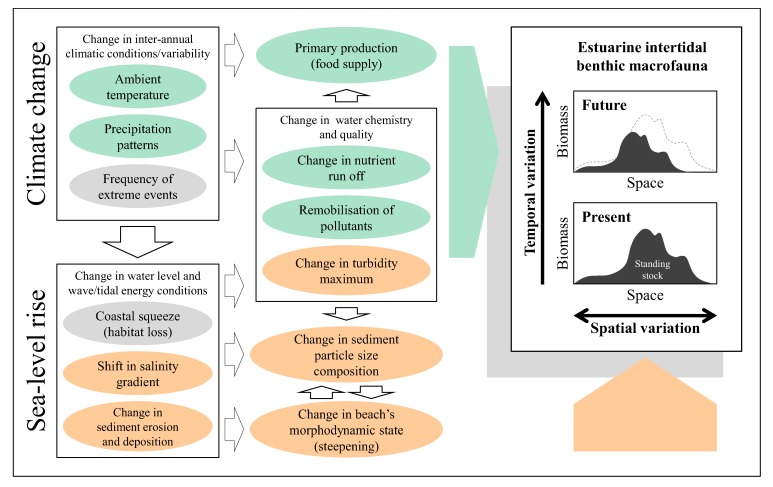
Conceptual representation of inter-relationships within/between broad environmental stressors induced by climate change and sea-level rise and spatio-temporal responses of estuarine intertidal macrobenthic biomass at the estuarine system scale. Stressors with respective pale green, orange and grey background represent environmental drivers which affect variability of macrobenthic biomass: (**1**) temporally (pale green); (**2**) spatially (orange); (**3**) both spatially and temporally (grey).

The above review indicates that benthic macrofauna plays a pivotal role in estuarine trophic interactions and the standing stock of macrobenthos is likely to be affected by a number of environmental parameters. Based on this, Figure 1 summarises possible inter-relationships between broad environmental changes/stressors induced by climate change and sea-level rise and spatio-temporal responses of intertidal macrobenthic biomass within a typical estuary. Climate change leads to changes in inter-annual climatic conditions such as ambient temperature, patterns of precipitation and frequency of extreme weather, whereas associated sea-level rise induces problems of coastal squeeze, upstream shift in salinity gradient and changes in patterns of sediment erosion and deposition, all of which in turn give rise to various other stressors and affect the spatio-temporal variability in the standing stock of benthic macrofauna both directly and indirectly in an estuarine ecosystem. At the whole estuary scale, temporal change in the total macrobenthic biomass available for higher trophic levels is affected by such factors as changes in ambient temperature, levels of nutrient supply and primary productivity (stressors with pale green background in Figure 1). Increased frequency of extreme weather conditions and habitat loss due to coastal squeeze may well interact adversely with various other stressors and affect variability in the standing stock of intertidal macrobenthos both spatially and temporally within an estuarine ecosystem over time (stressors in grey background in Figure 1). Impacts of sea-level rise will also be manifested through upstream shift in salinity gradient and changes in the pattern of sediment erosion and deposition which leads to changes in particle size distributions and beach’s morphodynamic states and hence spatial variation in the amount of macrobenthic biomass across the estuarine intertidal habitats (stressors in orange background in Figure 1).

**Figure 2:**
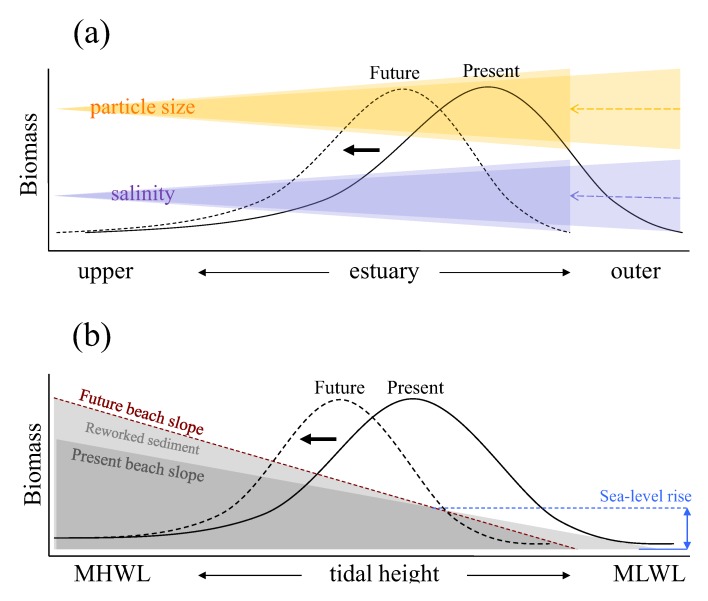
Schematic representation of relations between estuarine intertidal macrobenthic biomass and key environmental factors with conceptual models illustrating possible shifts in the biomass distributions in response to sea-level rise: (**a**) along estuarine longitudinal gradient with biomass shift due to salinity intrusion upstream and change in particle size distributions resulting from sea-level rise; (**b**) along estuarine vertical gradient over intertidal flat with biomass shift due to change in intertidal beach profile. The black solid and dotted lines represent present and future distributions of macrobenthic biomass, respectively. In diagram (**b**), the blue dashed line indicates increased sea level and the brown dashed line represents newly established intertidal profile in response to sea-level rise. MHWL and MLWL represent mean high water level and mean low water level, respectively.

Given uncertainties and the stochastic nature of some of the environmental stressors which primarily affect temporal aspect of estuarine macrobenthos, construction of deterministic models based on all the inter-relationships presented in Figure 1 to precisely describe future responses in macrobenthic biomass may be of substantial challenge. However, as has been reviewed in this study, there are a set of key environmental drivers that are amenable to empirical modelling and could therefore be used in the context of coastal and estuarine management in order to investigate the likely course of change in the amount of macrobenthic biomass in response to the expected physical changes induced by sea-level rise. Figure 2 shows schematic representation of relationships between such key environmental factors and spatial responses of macrobenthic biomass along estuarine longitudinal and vertical gradients based on empirical studies conducted in the Humber estuary UK [25,83].

Along the longitudinal gradient, present macrobenthic biomass per unit area is controlled mainly by salinity gradient and sediment particle size distribution (the black solid line in Figure 2a). Here, biomass is negatively affected by low salinity towards fresh water end and by the coarser sediment particle sizes which tends to accumulate near the mouth of the estuary. At this scale, sea-level rise is likely to influence macrobenthic biomass through the upstream shift in salinity gradient and sediment particle size redistributions. In response to such physical changes, the distribution of macrobenthic biomass is expected to shift along estuarine longitudinal gradient with area of high macrobenthic biomass (high quality intertidal area) relocated upstream in the future (the black dotted line in Figure 2a). Under such circumstances, the abundance of deposit feeders is likely to decline where the loss of fine particles is marked, but suspension feeders may increase in abundance particularly around the outer regions of estuaries, assuming that any increased load of sediment in suspension is not sufficient to reduce filter feeding efficiency. In addition, estuaries typically have larger intertidal areas towards the outer regions, and the upstream shift of macrobenthic biomass distribution indicated in Figure 2a suggests that the high quality area, which is currently located around the outer region, could become constrained by narrower intertidal area as the shift progresses towards up estuary, leading to a disproportionate reduction of total macrobenthic biomass at the estuarine system scale overall.

Along the vertical gradient from mean high to low water levels, present macrobenthic biomass per unit area is affected by the tidal height (the black solid line in Figure 2b) at any point of the longitudinal gradient. At this scale, sea-level rise will affect macrobenthic biomass through changes in beach profile or morphodynamic state resulting from increased wave energy and associated changes in the patterns of sediment reworking (the black dotted line in Figure 2b). For example, where the sea defence is located near to the high water mark, the intertidal area will be squeezed between the rising sea levels and the sea defence (Figure 2b). This would lead to a steepening and loss of intertidal area and hence loss of feeding area and amount of prey for higher trophic levels. Furthermore, sediment erosion may increase mainly through a worsening wave climate, and the fine material suspended would then be removed to low energy sinks elsewhere [84], which may reduce even further the quality of remaining intertidal flats. In addition, if upper parts of the beach are most affected, the recruitment of species, such as *Arenicola marina* and *Macoma balthica* which normally establish at higher shore levels, may be adversely affected. These scenarios indicate that some intertidal habitats are likely to experience loss of macrobenthos not only through the quantitative reduction in spatial area of intertidal habitats but also their qualitative reduction due to adverse shifts in local physical conditions in response to sea-level rise, and combination of such effects could lead to a disproportionate reduction of macrobenthic biomass than a straightforward loss of intertidal area would suggest in an estuarine ecosystem.

The relations in Figure 2 also indicate that there are nested structuring forces from whole system through longitudinal to local vertical scales, and the amount of prey biomass available for higher trophic levels should therefore be viewed differently depending on what scale is used in estuarine and coastal management. At the system wide scale in the Humber estuary, detailed model simulation study showed that a sea-level rise of 0.3 m would result in a 6.7% loss of intertidal area and 6.9% loss of total macrobenthic biomass, yet saline intrusion could partly compensate for such loss. However, associated environmental changes, such as beach slope steepening and changes in sediment distributions, could have overriding negative impacts with potential loss of macrobenthic biomass of up to 22.8%, depending on the extent and combination of environmental changes in response to sea-level rise [83]. Important implications from this study are that areas with high macrobenthic biomass are currently situated around the outer region of the estuary where extensive shallow muddy intertidal areas can be found, and such areas will also be the most subject to the impacts of sea-level rise due to their relatively exposed location and the shallowness of the beach slope. It follows therefore that effort needs to be made to identify suitable sites for habitat recreation around the outer rather than middle or upper region of the estuary if management is to effectively counteract the future loss of macrobenthic biomass.

In the case of estuaries with narrow, restricted entrances or with a bar, it is possible that a general increase in sediment supply could lead to accretion of material on intertidal flats as the suspended matter enters a less hydrodynamically energetic environment [84], as seen in many man-made harbours. The net effect here could be an increase in the elevation of the flat, depending on the relative rates of sea-level rise, sedimentation and land subsidence, reducing the productivity of the flats in the long term and even allowing conversion of flats to salt marsh. On the other hand, increases in ambient temperatures and milder winters, at least in Europe, together with enhanced primary production stimulated by increased nitrogen run off [85], may serve to increase invertebrate biomass and productivity and offset the negative effects of changes in sediment particle sizes and beach morphodynamic state, although it is very difficult to quantify such possibilities at present.

## 10. Conclusions

In typical European estuaries, 90%–95% of the estuarine intertidal habitats are bare mudflats and sandflats [4] that are important feeding and nursery grounds for the higher trophic levels, and it is therefore important to secure such habitats to counteract the future loss of macrobenthic prey resulting from sea-level rise, which should be built into the process of decision making in coastal and estuarine management. From an ecological point of view, one of the primary management objectives should be to maintain the current levels of standing crops of intertidal macrobenthos at the estuary system scale, and effective conservation measure would need to involve providing sufficient space to allow intertidal habitats to migrate and evolve in response to the changing environmental conditions. However, for many estuaries with low-lying hinterland, such transgression is unlikely to be permitted due to existing sea defence walls, and the most likely adaptive strategy selected by coastal managers to date has been to either “do nothing” where the land is undeveloped, or to reinforce existing sea defences (“hold the line” approach) in order to reduce the risk of flooding particularly in front of existing developed urban or industrial areas or high-grade agricultural lands. In the face of the expected rate of sea-level rise in the future, however, “managed realignment” has gradually gained acceptance as a preferred option in the context of sustainable coastal and estuarine management in the UK as well as in other part of the world [86,87]. Managed realignment aims to recreate saltmarsh and mudflats by setting back existing sea defence wall further inland and allowing the created area to be tidally inundated, which could potentially compensate for the future loss of intertidal habitats and ecological integrity of estuarine environments resulting from sea-level rise. However, this option still remains contentious and has not been adopted on a large scale. Even where the proposals for realignment have been taken forward, such schemes have been often based on opportunistic grounds, for example, in response to an accidental defence failure [87]. Furthermore, economically viable coastal defence is the primary objective for such schemes, and managed realignment is thus considered primarily to improve both coastal stability and cost performance by replacing costly artificial “hard” coastal protection with less costly natural “soft” coastal landforms. For this reason, main focus in the implementation of managed realignment has been concentrated on the re-creation of “salt marshes” in order to provide the expansive energy dissipation system [87,88,89], and none of the schemes have yet made an attempt to minimise the negative ecological impacts addressed in this study. Intertidal mudflats and sandflats within an estuary exhibit significant spatial variations in the biomass of macrobenthic prey along key environmental gradients and such knowledge can be readily used when prioritising site selection and calculating the sizes necessary for habitat recreation in the management planning process. For example, one of the intertidal mudflats located in the upper region in the Humber estuary had a mean macrobenthic biomass of 0.04 g AFDW m^−2^, whereas other extensive mudflat of Spurn Bight situated at the outer region of the Humber showed an average biomass of 17.1 g AFDW m^−2^. This indicates that creation of 400 ha intertidal habitat in the upper region by implementing managed realignment could not even compensate for the loss of 1 ha of mudflat in Spurn Bight in the Humber in terms of securing total macrobenthic biomass available for higher trophic levels at estuarine system scale. Replacement sites must therefore be identified and their appropriate size must be calculated on the basis of a sound ecological understanding of estuarine ecosystems if we are to counteract the future loss of intertidal habitats and to maintain the current levels of macrobenthic biomass through the implementation of such restoration schemes. 

A further consideration is that any delay of implementing appropriate adaptive strategies in the coastal region will reduce their effectiveness in reducing ecological and socio-economic impacts over the medium and long term, when the effects of sea-level rise could be catastrophic. On a regional scale, there is already increasing evidence that intertidal habitats is being lost at alarming rate as a result of rising sea-levels coupled with coastal subsidence. For example, almost 60% of wetland loss observed along the northern Gulf of Mexico was due to the net effect of sea-level rise and subsidence [90], and along the Louisiana coast, annual losses of up to 73 km^2^ of wetland area were attributable to the increased rate of relative sea-level rise during the 20th century [91].

Finally, sea-level rise is just one of many stressors which will affect estuarine environments in the future and it is by no means clear whether the combined impacts of different stressors can be simply treated as additive. If they are non-additive in their impacts, then a small change in sea-level rise may exert an impact greater than expected. Further studies are needed to increase certainty in predicting how estuaries are likely to change and interact with contemporary climate change and associated rising sea levels in order to prevent any one of the stressors from triggering catastrophic impacts on the ecology of intertidal habitats, which may in turn cause widespread impacts on heavily estuarine dependent higher trophic levels including humans.
